# Data-driven statistical modeling of the emergent behavior of biohybrid microrobots

**DOI:** 10.1063/1.5134926

**Published:** 2020-02-28

**Authors:** Eric J. Leaman, Ali Sahari, Mahama A. Traore, Brian Q. Geuther, Carmen M. Morrow, Bahareh Behkam

**Affiliations:** 1 Department of Mechanical Engineering, Virginia Tech, Blacksburg, Virginia 24061, USA; 2 School of Biomedical Engineering and Sciences, Virginia Tech, Blacksburg, Virginia 24061, USA

## Abstract

Multi-agent biohybrid microrobotic systems, owing to their small size and distributed nature, offer powerful solutions to challenges in biomedicine, bioremediation, and biosensing. Synthetic biology enables programmed emergent behaviors in the biotic component of biohybrid machines, expounding vast potential benefits for building biohybrid swarms with sophisticated control schemes. The design of synthetic genetic circuits tailored toward specific performance characteristics is an iterative process that relies on experimental characterization of spatially homogeneous engineered cell suspensions. However, biohybrid systems often distribute heterogeneously in complex environments, which will alter circuit performance. Thus, there is a critically unmet need for simple predictive models that describe emergent behaviors of biohybrid systems to inform synthetic gene circuit design. Here, we report a data-driven statistical model for computationally efficient recapitulation of the motility dynamics of two types of *Escherichia coli* bacteria-based biohybrid swarms—NanoBEADS and BacteriaBots. The statistical model was coupled with a computational model of cooperative gene expression, known as quorum sensing (QS). We determined differences in timescales for programmed emergent behavior in BacteriaBots and NanoBEADS swarms, using bacteria as a comparative baseline. We show that agent localization and genetic circuit sensitivity strongly influence the timeframe and the robustness of the emergent behavior in both systems. Finally, we use our model to design a QS-based decentralized control scheme wherein agents make independent decisions based on their interaction with other agents and the local environment. We show that synergistic integration of synthetic biology and predictive modeling is requisite for the efficient development of biohybrid systems with robust emergent behaviors.

## I. INTRODUCTION

Microrobots defined as autonomous or semi-autonomous systems with characteristic dimensions O (1 *μ*m) were envisioned as valuable non-invasive tools for medical intervention well before they became technologically feasible.^1^ Recent decades have seen a myriad of microrobotic concepts and prototypes developed, mainly for medical applications.^2,3^ The most significant challenges in developing such systems are incorporating effective mechanisms for actuation, sensing, and control, all without the need for an onboard power source. Repeatedly, biology has offered great solutions to these challenges both as a source for design inspiration^4^ and, more prominently, by the incorporation of biological materials themselves as part of the microrobotic systems.^5^ Designs have incorporated eukaryotic cells or unicellular organisms, such as algae,^6^ spermatozoa,^7^ macrophages,^8^ and cardiomyocytes^9,10^ but more commonly have relied upon swimming bacteria as actuators.^11,12^ Bacteria efficiently transduce chemical energy from their environment into kinetic energy for self-propulsion and possess robust mechanisms to sense a wide range of environmental stimuli including chemical, optical, thermal, or magnetic.^13–18^ These characteristics, along with their small physical dimensions, O (1 *μ*m), and their ability to tolerate changes in temperature, pH, nutrient availability, and other environmental conditions have made bacteria an ideal candidate for building biohybrid microrobotic systems and have been investigated for such applications for over 15 years.^19–22^ Pioneering bacteria-based microrobotic works include development of a bacteria-based microassembly system by Martel *et al.*,^21^ using *Magnetospirillum gryphiswaldense* to position microparticles under the control of an external magnetic field. One of the earliest works demonstrating bacteria-based drug delivery, a primary application of microrobots, was the use of *Listeria monocytogenes* to transfer drug-loaded nanoparticles into the cytoplasm of cancer cells by Akin *et al.*, taking advantage of the bacteria's natural propensity for cellular invasion.^23^ In the same year, Behkam and Sitti^20^ developed a chemical switching technique for on-demand stop/go control of the motility of *Serratia marcescens*-propelled polystyrene microparticles by turning the flagellar motors off and on through introduction of copper ions and subsequent sequestration of the ions via ethylenediaminetetraacetic acid (EDTA).

The sensing capabilities of bacteria make centralized control via externally imposed stimuli gradients an attractive microrobotic system design paradigm. Indeed, centralized directional control of bacteria-based biohybrid systems has been achieved utilizing the wide array of bacterial taxis mechanisms for gradient sensing. Martel and colleagues have extensively developed magnetotactic bacteria-based microrobots that sense and respond to magnetic fields for particle placement,^21^ complex assembly,^24^ and drug delivery, including an *in vivo* demonstration.^25^ In addition to utilizing native biological mechanisms, magnetic-field based directional control has been achieved using the model motile bacteria *Escherichia coli* assembled with erythrocytes that were loaded with magnetic nanoparticles and doxorubicin^26^ and swimming algae with conjugated magnetic nanoparticles.^27^ Gradients of chemoattractants have been utilized to directionally control a variety of bacteria-based biohybrid systems, which was first demonstrated when our group used casamino acids to bias the migration of spherical polystyrene microparticles decorated with *S*. *marcescens.*^28^ Since then, we and others have shown that chemotaxis in bacterial biohybrid systems in response to several chemoeffectors,^29–31^ can be used to control microrobotic systems such as *E. coli*-propelled microparticles of various geometries,^32^ *E. coli* coated with nanoparticles,^33^ and others.^31,34,35^ Other centralized control mechanisms reported include the use of UV light,^22^ pH gradients,^36^ and electric field gradients.^37^

The small size and limited capabilities of an individual microrobotic agent necessitate the cooperation of a large number of agents, often swarms of hundreds or more, to accomplish a given task. Centralized control approaches can be effective in controlling populations, but individual addressability of each agent is limited, precluding the ability to achieve fully deterministic outcomes. For instance, chemotactic control of microrobot migration cannot realize perfect sorting; only a fraction of the population will respond.^33^ Centralized control approaches that allow individual addressability, such as using electric field actuation control and UV light for individual steering, are more robust; however, scalability to large populations may be limited.^38^ Robust and scalable outcomes could be achieved by implementing hybrid control strategies that combine a centralized control scheme with a decentralized one. A decentralized control scheme would allow each agent to make independent decisions based on their interaction with other agents and their local environment. For instance, in a drug delivery application, a decentralized control mechanism may be utilized to minimize off-target cargo release by the agents that failed to respond to the centralized control signal to reach the site of interest. This way, the agents will autonomously become activated and perform their desired task only if a large fraction of the swarm reaches the site of interest. Lagging agents would either never become activated or only later become activated upon successfully reaching the site of interest.

The burgeoning field of synthetic biology allows for the engineering of programmed behavior in eukaryotic and prokaryotic cells^39^ and has vast potential benefits for building biohybrid microrobotic swarms with sophisticated centralized and decentralized control schemes. One particularly powerful genetic circuit paradigm is quorum sensing (QS), which is a number-density dependent form of population cooperation. In QS organisms, small diffusible signaling molecules (hereafter referred to as the signal) are produced constantly at a low basal rate.^40^ Regulation of the enzyme that produces the signal is controlled by a positive-feedback loop with a signal-activated transcription factor. The circuit is bistable, and thus upon being exposed to a critical amount of signal (i.e., a minimum concentration threshold for a sufficient period of time), the circuit exhibits a switch-like behavior to an “activated” state of high signal enzyme production, as well as the transcription of any other genes downstream of the QS promoter. This mechanism has been extensively used for engineering bacterial populations to achieve programmed cell death,^41^ directional control,^42^ and advanced cancer therapies,^43^ including cytotoxin release for chemotherapy,^44,45^ and to deliver immunotherapeutic nanobodies.^46^

Synthetic biology is being increasingly integrated with biohybrid microrobotics to achieve new capabilities. We recently demonstrated that synthetic QS circuits can be used to address the need for engineering decentralized emergent behaviors in populations of biohybrid microrobots.^47^ Other functions, including engineering active biohybrid materials,^48^ creating biohybrid microrobotic sensors,^49^ and light-based control of cargo release by microrobots,^50^ have been reported. It should be noted that the design of synthetic circuits tailored toward specific functional property and performance characteristics is iterative in nature.^39^ Engineered circuits are traditionally characterized in small volumes of spatially homogeneous cell suspensions (i.e., in well plates), whereas biohybrid microrobotic systems are dynamic in nature and often distribute heterogeneously in complex environments with diverse transport boundary conditions. Thus, there is a critically unmet need for predictive models that describe the programmed emergent behavior of biohybrid systems to complement the standard experimental characterization of the engineered cells. The synergistic combination of the two approaches will streamline the development of biohybrid machines with robust and predictable emergent behaviors. Existing models of biohybrid microrobots either involve computationally intensive calculations of forces at the individual agent-scale^28,51–53^ or are carried out on the population-scale^54^ and unable to accurately capture the effects of motility on QS activation at the low agent concentrations relevant for biomedical applications.

**FIG. 1.**
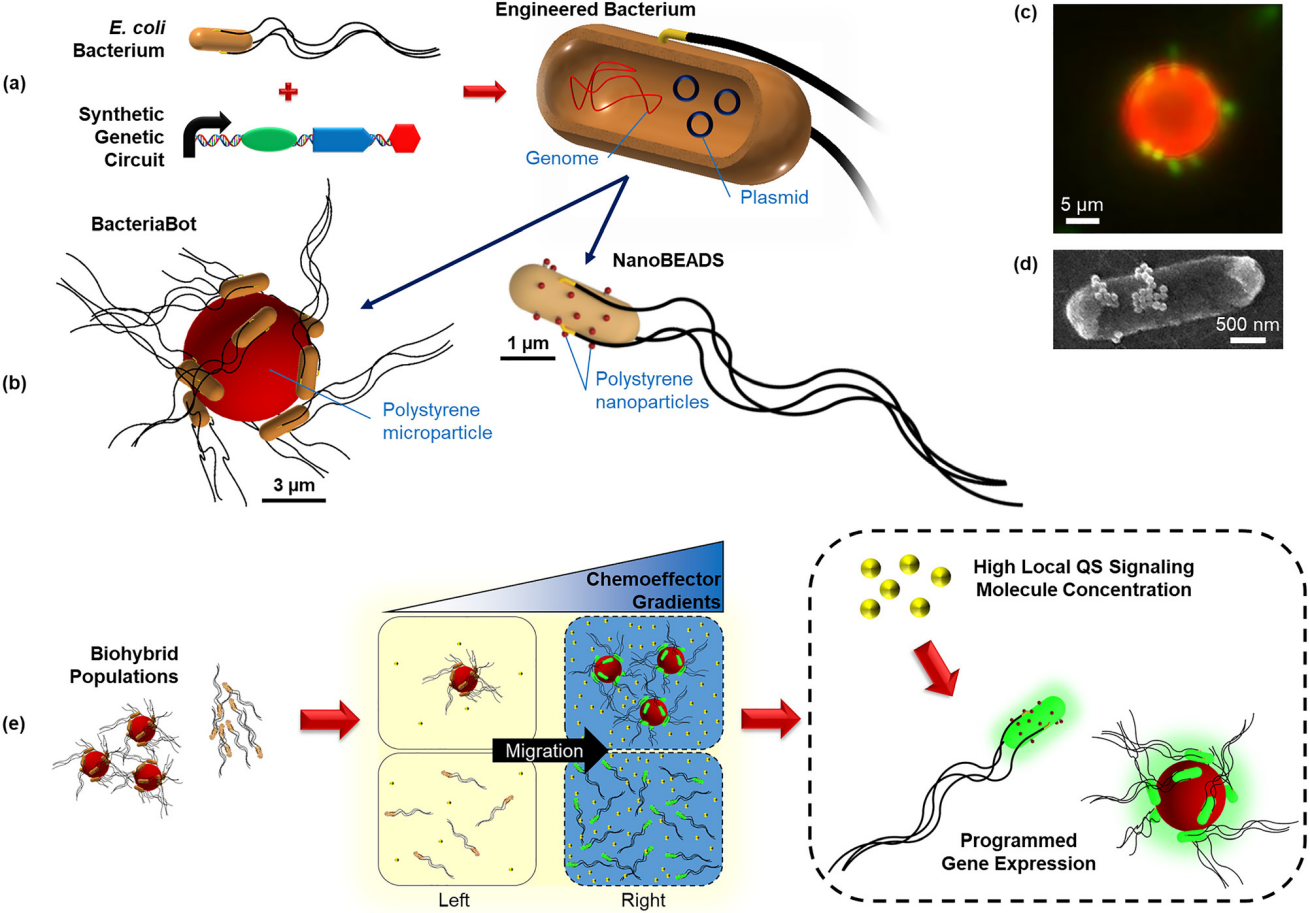
BacteriaBots and NanoBEADS biohybrid drug delivery agents. (a) *E. coli* bacteria are engineered with synthetic genetic circuits inserted into plasmids; (b) the engineered bacteria are integrated into biohybrid systems to make BacteriaBots (6 *μ*m polystyrene particles conjugated with several bacteria) and NanoBEADS (bacteria coated with several polystyrene nanoparticles). Representative microscopy images of a BacteriaBot and a NanoBEADS agent are shown in (c) and (d), respectively. (e) The engineered biohybrid systems are capable of responding to centralized control signals (chemoeffector gradients) and commencing population density dependent programmed behavior upon detecting a high enough local concentration of QS signaling molecules (decentralized control).

In this paper, we aim to address this gap through the development of a computationally efficient stochastic model that recapitulates motility, chemotaxis, and QS in two types of biohybrid bacteria-based agents, BacteriaBots (BB)^55^ and NanoBEADS (NB),^56^ both of which were developed for controlled transport of cargo (e.g., drug delivery) applications. The agents are fundamentally different in design; as a result, they differ significantly from one another in motile and cooperative behaviors. A BacteriaBot consists of 6 *μ*m-diameter spherical particles conjugated with 8–15 *E. coli* bacteria, while a NanoBEADS agent consists of a bacterium conjugated with an average of 20 nanoparticles on the outer membrane [Figs. 1(a)–1(d)]. We report an agent-based computational model that directly utilizes experimental data to simulate the motile behavior and spatial distribution of the agents, which is then used to predict the emergent behavior of QS in BacteriaBots and NanoBEADS [Fig. 1(e)]. We show that by collecting limited experimental data on agent motility in a chemically isotropic environment (i.e., in the absence of a chemoattractant gradient), we are able to closely recapitulate the experimental migration bias in response to a chemoattractant gradient in both space and time. Our method is simple, fast, and generalizable to any type of biohybrid system with random motility. Here, we use our model to explore differences between the timescales for emergent behavior in populations of BacteriaBots and NanoBEADS, using free-swimming bacteria as a comparative baseline. We then explore the sensitivity and robustness of each system across the QS genetic circuit design space with respect to migration bias. Finally, we show that QS-based decentralized control can be an effective mechanism for causing the desired activation within a target site among spatially separated subpopulations but that results depend critically on the agent type, demonstrating the crucial role of computational modeling in the design of biohybrid microrobots with robust emergent behavior.

## II. RESULTS AND DISCUSSION

### A data-driven model of biohybrid microrobot motility and chemotaxis

A.

**FIG. 2.**
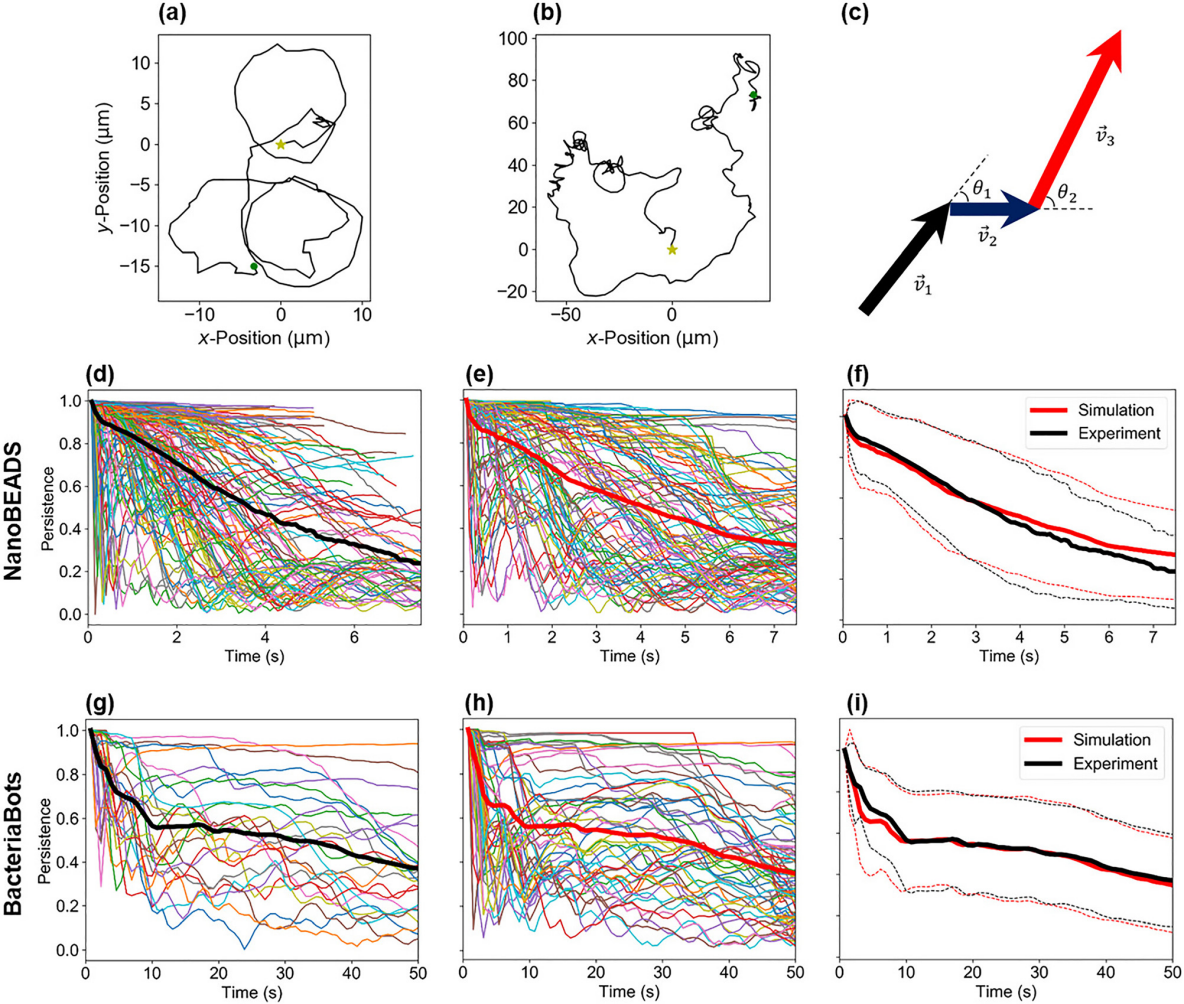
The data-based statistical motility model accurately recapitulates NanoBEADS and BacteriaBots swimming behavior. Representative trajectories for a NanoBEADS agent (a) and a BacteriaBot (b) produced from the experimental data (yellow stars represent the starting position while green dots represent the final position of the tracked agents), (c) a schematic of the successive agent position vectors and orientation change, persistence as a function of time for each tracked (d) and simulated (e) NanoBEADS agent, (f) persistence vs time averaged over tracked and simulated NanoBEADS agents, persistence vs time for each tracked (g) and simulated (h) BacteriaBot agent, and (i) persistence vs time averaged over tracked and simulated BacteriaBots agents. A total of 154 NanoBEADS and 26 BacteriaBots agents were tracked from experiments, and all tracked data were collected in isotropic chemical environments. Thick red and black curves in (d)–(i) indicate average persistence. The red and black dashed traces in (f) and (i) indicate ±standard deviation for simulated and experimental data, respectively.

The primary goal of the computational framework developed herein is to enable computationally efficient recapitulation of the dynamics of swarms of BacteriaBots and NanoBEADS, toward the predictive modeling of their emergent behavior such as a QS response. The ability to faithfully recapitulate motility and chemotaxis is crucial as QS inherently depends upon local agent concentration and transport properties. We aimed to establish a computationally efficient and straightforward model that can be quickly adopted by researchers of diverse backgrounds in the biohybrid machines community. To this end, we conducted a series of motility assays and utilized the experimental data in our simulations, as an alternative to building a force-based model that would be a more complex and computationally intensive approach. We accomplished this by recording time-lapse images of motile BacteriaBots and NanoBEADS in chemically isotropic environments and tracking the positions of the agents over time [Figs. 2(a) and 2(b)]. The velocity vector between every two consecutive data points was calculated, providing the speed and orientation of each agent with respect to time [Fig. 2(c)]. The average speed and the time rate of orientation change of BacteriaBots were 2.82 ± 1.62 *μ*m/s and 7.96 ± 70.0 deg/s, respectively. NanoBEADS swam substantially faster with an average speed of 23.4 ± 10.0 *μ*m/s and a rate of orientation change of 62.7 ± 606 deg/s. For isotropic simulations, each agent randomly selected the trajectory data of a tracked experimental agent, capturing its motile behavior by sampling from its speeds and rates of orientation change in sequence at time intervals consistent with experimental data acquisition. Once a simulated agent utilized the entire duration of data for a selected experimental agent, the data for another experimental agent were randomly chosen, creating continuous trajectories for the duration of the simulation.

**FIG. 3.**
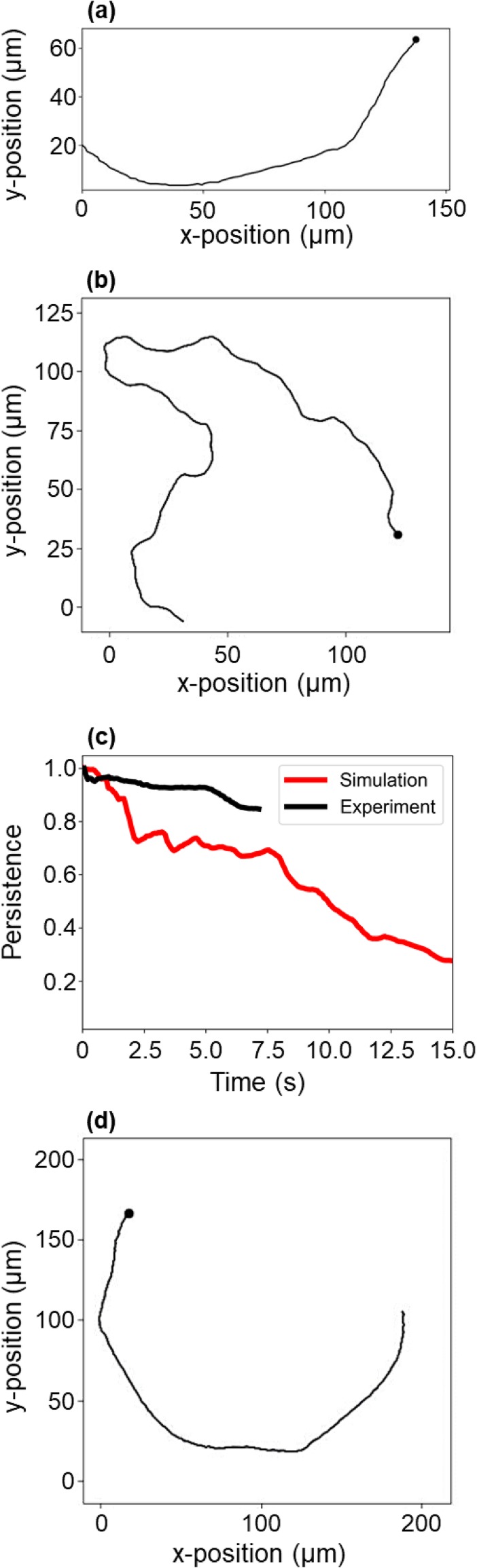
Data sampling in-sequence is required to recapitulate experimental agent motile behavior. (a) The spatial trajectory of a representative highly persistent NanoBEADS agent experimental agent, (b) the spatial trajectory of an agent simulated by random sampling, (c) persistence vs time for a single agent (red curve) by random sampling from a single tracked experimental agent (black curve), and (d) the spatial trajectory of the agent by sampling in sequence from the data for the same experimental agent. Note that once all experimental data have been sampled (without replacement), all data are reset to permit simulations of indefinite duration.

We used persistence as a metric by which to compare the motile behavior of simulated and experimental agents,

$$P\left(t_{N}\right)=\frac{\mathrm{displacement}}{\mathrm{distance}}=\frac{\sqrt{{\left(x_{N}-x_{0}\right)}^{2}+{\left(y_{N}-y_{0}\right)}^{2}}}{\sum_{i=2}^{N}\sqrt{{\left(x_{i}-x_{i-1}\right)}^{2}+{\left(y_{i}-y_{i-1}\right)}^{2}}},$$ (1)

where t is time, N is the number of time steps, and x and y are an agent's coordinates. As shown in Fig. 2(d), the persistence of NanoBEADS in time varied widely, with some agents being highly persistent over the entire duration of a trajectory and others moving at very low persistence. The persistence of BacteriaBots [Fig. 2(g)] was much higher than that of NanoBEADS, particularly when the timescale of tracked data is considered. Simulations of each agent type captured both the distributions [Figs. 2(e) and 2(h)] and the averages [Figs. 2(f) and 2(i)] of persistence values in time. It is important to note that capturing the persistence distribution required our method of sampling speed and rate of orientation change in sequence. Randomly sampling from the values extracted for a highly persistent agent did not capture the essence of that agent's trajectory [Figs. 3(a) and 3(b)]. This resulted in persistence values of the simulated agent much lower than those of the experimental agent [Fig. 3(c)], while sampling in sequence well captured the trajectory *in silico* [Fig. 3(d)]. Overall, random sampling failed to produce the subpopulation of highly persistent agents observed experimentally (Fig. S1).

**FIG. 4.**
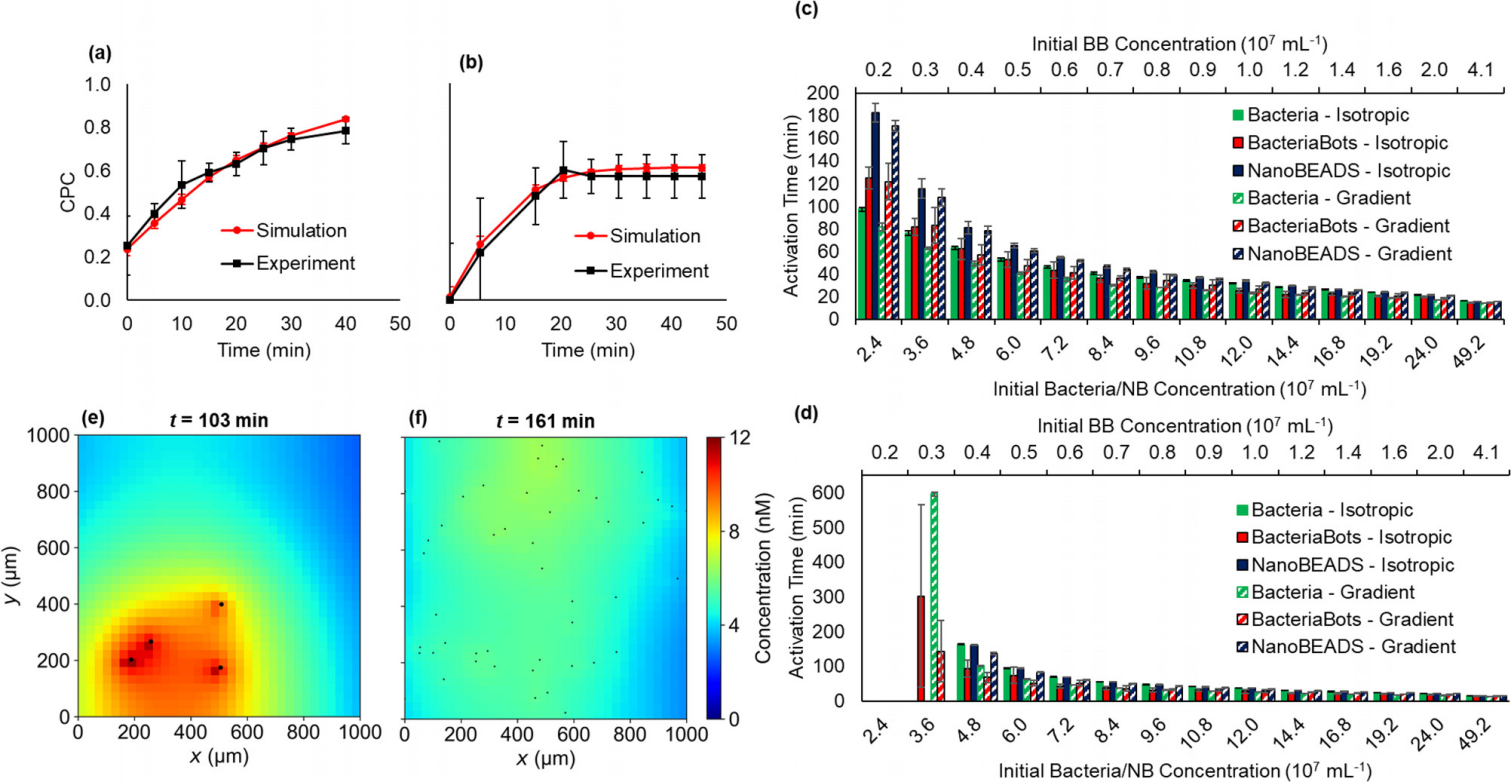
The emergent behavior of populations of BacteriaBots (BB) and NanoBEADS (NB). Experimental and simulated CPC as a function of time for BacteriaBots (a) and NanoBEADS (b). QS activation time as a function of the concentration of BacteriaBots or equivalent total number of free-swimming bacteria without (isotropic) or with (gradient) an imposed chemoattractant gradient with experimentally measured doubling times of 43 min for free-swimming bacteria and 121 min for NanoBEADS and BacteriaBot-conjugated bacteria (c) and without growth (d). (e) and (f) respectively show snapshots of the QS signal concentration field and agent positions at the QS activation time in simulations of BacteriaBots at a concentration of 0.4 × 10^7^ ml^−1^ and the corresponding concentration of 4.8 × 10^7^ ml^−1^ NanoBEADS, both in isotropic environments.

In order to simulate responses to linear chemoattractant gradients (i.e., chemotaxis) across a rectangular domain, we randomly chose two equally sized subsets of experimental trajectories—one from which simulated agents moving in the negative x-direction (at the start of a new trajectory) were sampled and one from which simulated agents moving in the positive x-direction were sampled. In this way, the simulated agent movement could be biased due to the net migration bias present in the separated experimental data. This process was repeated iteratively (i.e., randomly creating new subsets each time) until the simulated spatial distribution of the agents matched the experimental data collected at the chemoattractant gradient that produced the maximum population distribution bias (Sec. IV A). The chemotaxis partition coefficient (CPC) was used as a metric for comparing bias in the swarm spatial distribution due to chemotaxis:

$$CPC=\frac{B_{R}-B_{L}}{B_{R}+B_{L}},$$ (2)

where $B_{L}$ and $B_{R}$ are the numbers of agents in the left-hand half and the right-hand half of the domain, respectively [Fig. 1(e)]. As described above, the simulated CPC was produced through the iterative determination of particular separations of the experimental trajectory data that led to strong matches between simulated and experimental chemotaxis responses for both BacteriaBots [Fig. 4(a)] and NanoBEADS [Fig. 4(b)] as a function of time.

Our unique method of directly utilizing isotropic experimental data for simulations of motility and chemotaxis has several distinct advantages. Most important for the purposes of our model, it is guaranteed to accurately capture the speed, rate of orientation change, and persistence of actual agents. It is far more computationally efficient than resolving forces generated on a microparticle in the case of BacteriaBots^28^ or than modeling the drag forces acting on a bacterial cell due to attached nanoparticles in the case of NanoBEADS.^56^ Moreover, the method is generalizable to any motile agent and could be quickly implemented by any researcher. Finally, only a relatively limited amount of tracking data is needed to impart the ability to simulate agents moving isotropically or in a particular biased manner, as illustrated by our matching of CPC as a function of time using 26 BacteriaBot trajectories.

### B. Engineering QS-based cooperative behavior in bacterial biohybrid systems

We used our data-driven motility and chemotaxis model in conjunction with our experimentally validated model of cell–cell communication and QS response^47,57^ to investigate the timeframe for QS-based emergent behavior to commence (hereafter referred to as the QS activation time) in populations of NanoBEADS and BacteriaBots. For this work, QS activation time was determined by finding the point in time when the average intracellular concentration of QS-controlled green fluorescent protein (GFP) surpassed a detectable threshold (218 molecules/bacterium), but in practice, the gene for GFP expression may be replaced with another to produce desirable behavior (e.g., synthesis of cytotoxic drugs for cancer therapy). For each simulation, we considered a 1000 × 1000 *μ*m^2^ domain with periodic y-boundaries and zero-concentration x-boundaries placed at a distance of 1000 *μ*m from the 1000 × 1000 *μ*m^2^ motility domain. QS activation time in engineered free-swimming bacteria was also computed to provide a baseline for comparison. For simulating free-swimming bacteria, we used our previously validated run-and-tumble and chemotaxis models.^57^

We first investigated activation time at biomedically relevant agent concentrations, both in chemically isotropic environments and in the presence of optimum chemoattractant gradients. Given that attenuated bacteria have been reported to colonize tumors at concentrations on the order of 10^8^ bacteria/cm^3^ (Refs. 58–61) and assuming a tissue density of 1 g/cm^3^, we simulated bacteria concentrations ranging from ∼2 × 10^7^ ml^−1^ to 50 × 10^7^ ml^−1^ (corresponding to 20–500 simulated bacteria). For all simulations, we compared equivalent numbers of total bacteria; therefore, any given concentration of NanoBEADS was compared with BacteriaBots at 1/12th the concentration, as an average of 12 bacteria are attached to a particle for each BacteriaBot [Figs. 1(b) and 1(c)]. In addition to biohybrid agents, we also simulated free-swimming bacteria (speed of 34.0 *μ*m/s) as a comparative baseline using our previously developed stochastic motility and chemotaxis models.^57^ The doubling time of bacteria was taken to be 43 min for free-swimming bacteria and 121 min for NanoBEADS and bacteria attached to microparticles (Sec. IV D), leading to shorter activation times for bacteria in low concentration scenarios (when the timescale for activation was long enough to allow for an appreciable amount of growth). In isotropic cases at an initial concentration of 2.4 × 10^7^ ml^−1^ for NanoBEADS and 0.2 × 10^7^ ml^−1^ for BacteriaBots, respectively, 88% and 28% more time was required for the agents to become activated, compared with free-swimming bacteria [Fig. 4(c)]. The differences were greater with simulated chemotaxis, with NanoBEADS and BacteriaBots having 110% and 49% longer activation times than bacteria, respectively. Overall, differences between activation times of the different agent types were most pronounced at low concentration. At very high concentrations (>24 × 10^7^ ml^−1^), activation times converged regardless of the agent type and migration bias. Chemotaxis had virtually no effect on the activation time of BacteriaBots and only a small relative effect on NanoBEADS (average 5% increase in activation time for isotropic cases up to 24 × 10^7^ ml^−1^). However, chemotaxis had a moderate effect on the activation time for free swimming bacteria at low concentrations, causing a 30% delay on average in simulated isotropic cases relative to chemotaxis cases for initial concentrations up to 24 × 10^7^ ml^−1^. This is because bacteria become highly localized more quickly than NanoBEADS or BacteriaBots in the chemotactic scenarios, reaching steady state CPCs of approximately 0.95 after only 5 min. In contrast, it took NanoBEADS 20 min to reach a steady state CPC of approximately 0.6 [Fig. 4(b)]. For BacteriaBots, chemotaxis played almost no role because for the same number of bacteria, there were many fewer individual agents (average of 12 bacteria per BacteriaBot), making the role of stochastic movement more significant than that of an emergent population-scale distribution bias.

In addition to simulating scenarios with growth, it is important to consider situations in which growth would not occur (e.g., nutrient-limited environment) or bacteria may be eliminated from the region of the interest (e.g., due to immune response) such that the population size will remain largely unchanged. As shown in Fig. 4(d), activation time was greatly affected by the lack of growth, particularly at concentrations below 16.8 × 10^7^ ml^−1^ (1.4 × 10^7^ ml^−1^ for BacteriaBots). Activation did not occur below a concentration of 4.8 × 10^7^ ml^−1^ for NanoBEADS and only with a chemical gradient for free-swimming bacteria but did occur for BacteriaBots at an equivalent concentration of 0.3 × 10^7^ ml^−1^. At a higher concentration of 4.8 × 10^7^ ml^−1^ (0.4 × 10^7^ ml^−1^ BacteriaBots), QS activation of free-swimming bacteria and NanoBEADS was comparable but about 70% longer than BacteriaBots in isotropic cases. This is due to the high local concentration of bacteria constrained on the surface of each BacteriaBot agent. The positive feedback behavior of the genetic circuit causes BacteriaBot behavior to be more stochastic than that of NanoBEADS at comparable concentrations, as moving near to one another for a short period of time once a sufficient level of background QS signal has accumulated facilitated activation [Fig. 4(e)]. This is less likely to occur with NanoBEADS since many (×12 more) individual agents are more dispersed in the analogous case [Fig. 4(f)]. As the concentration increased, activation times converged to similar values but remained shorter for BacteriaBots relative to bacteria in all isotropic cases and shorter relative to NanoBEADS in all cases. Interestingly, the activation time for free bacteria was longer than that of corresponding NanoBEADS simulations in all isotropic cases but shorter in all gradient cases. Differences between the isotropic cases are likely due to the lower speed of NanoBEADS (average of 23.4 *μ*m/s) relative to free-swimming bacteria (34.0 *μ*m/s), while faster activation of the bacteria in gradient cases is due to their stronger chemotaxis response. These results highlight the role of spatial distribution of agents in the temporal evolution of the QS response and the importance of accurately modeling motility and chemotaxis to faithfully recapitulate temporal changes in the spatial distribution of agents.

### C. Investigating system sensitivity and robustness

**FIG. 5.**
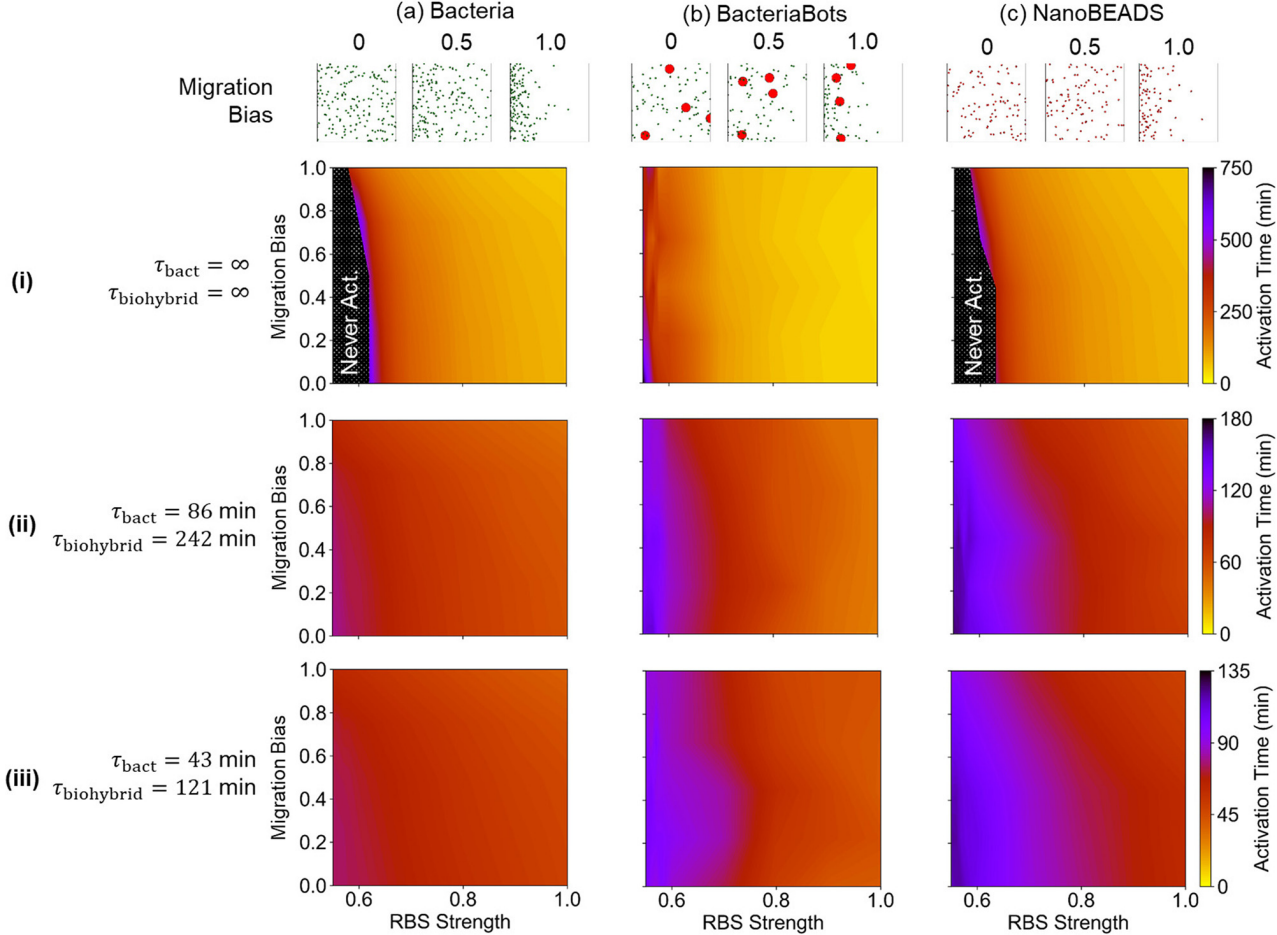
Emergent behavior across circuit sensitivity design space. Simulated activation time as a function of the chemotaxis response (migration bias) and RBS strength (circuit sensitivity) for Bacteria (a), BacteriaBots (b), and NanoBEADS (c) without growth (i), at half the experimentally measured growth rates (ii), and at the experimentally measured growth rates (iii). Representative distributions of agents (and free bacteria resulting from growth) at various migration bias after ∼100 min of simulation are shown for each agent type. Black shaded regions in (a-i) and (c-i) indicate zones where the agents reached a steady non-activated state.

One of the primary design considerations in engineering microrobotic swarms cooperating through QS is the sensitivity and robustness in their response. From a synthetic biology standpoint, the sensitivity of the QS genetic circuits (i.e., the amount of signal needed to cause the system to transition from its non-activated to its activated state) can be modulated across a wide range by altering the strength of the ribosomal binding site (RBS) for the *luxI* gene.^57^ As circuit sensitivity is increased, fewer agents and less time are needed to activate the circuit. This provides a valuable mechanism by which synthetic bacteria-based systems may be designed in practice to achieve target activation times. We have previously shown that we can accurately model the effects of changes in QS genetic circuit sensitivity on QS activation time.^57^ Nevertheless, uncertainties in key parameters affecting system performance, such as the growth rate, chemotaxis, mass transport boundary conditions, and environmental transport properties, among many others, will undoubtedly be present in any practical situation with the potential for deleterious effects on performance. Thus, in addition to the sensitivity, robustness in the response of the engineered systems should also be considered. We used our model to explore the sensitivity and robustness of the design space for BacteriaBots and NanoBEADS with respect to migration bias, using free-swimming bacteria as a comparative baseline. Experimental trajectory data were binned to provide steady-state CPC values for biohybrid agents ranging from 0 to approximately 0.9. Likewise, we imposed l-aspartic acid gradients to provide the same CPC range in free-swimming bacteria cases. These l-aspartic acid gradients or sets of binned trajectory data were ranked from weakest to strongest resulting CPC and defined as migration bias, ranging from 0 (corresponding to a CPC of 0) to 1 (corresponding to a CPC of ∼0.9). We then simulated each system for a biomedically relevant initial bacterial concentration of 4.8 × 10^7^ ml^−1^ (0.4 × 10^7^ ml^−1^ BacteriaBots) across a RBS design space, which ranges from the lowest relative RBS strength that permitted activation of at least one agent type with no growth to the reference strength.^57^ Simulations were performed with no growth, growth at half the experimentally measured rates, and growth at the experimentally measured rates (Fig. 5). The sensitivity in QS response, assessed by quantitating the QS activation time, was investigated as a function of the migration bias and RBS strength. Interestingly, BacteriaBots proved to be the most sensitive in the absence of growth [Fig. 5(b-i)], as activation occurred at RBS strengths as low as 0.55. In contrast, simulations at the same RBS strengths predicted large zones where activation did not occur for both bacteria [Fig. 5(a-i)] and NanoBEADS [Fig. 5(c-i)]. Moreover, BacteriaBot sensitivity was largely unaffected by the migration bias, even in scenarios with growth [Figs. 5(b-ii) and 5(b-iii)], while NanoBEADS activation time was affected by the migration bias, particularly in scenarios with growth [Figs. 5(a) and 5(c)]. This is because BacteriaBots' lower motility speed and unchanged proximity of the attached bacteria on each agent effectively concentrate QS signal generation in a small space. The nature of the QS regulatory circuit, i.e., its positive feedback-based self-regulation, allows BacteriaBots to become more robustly activated in scenarios when free bacteria and NanoBEADS may not.

**FIG. 6.**
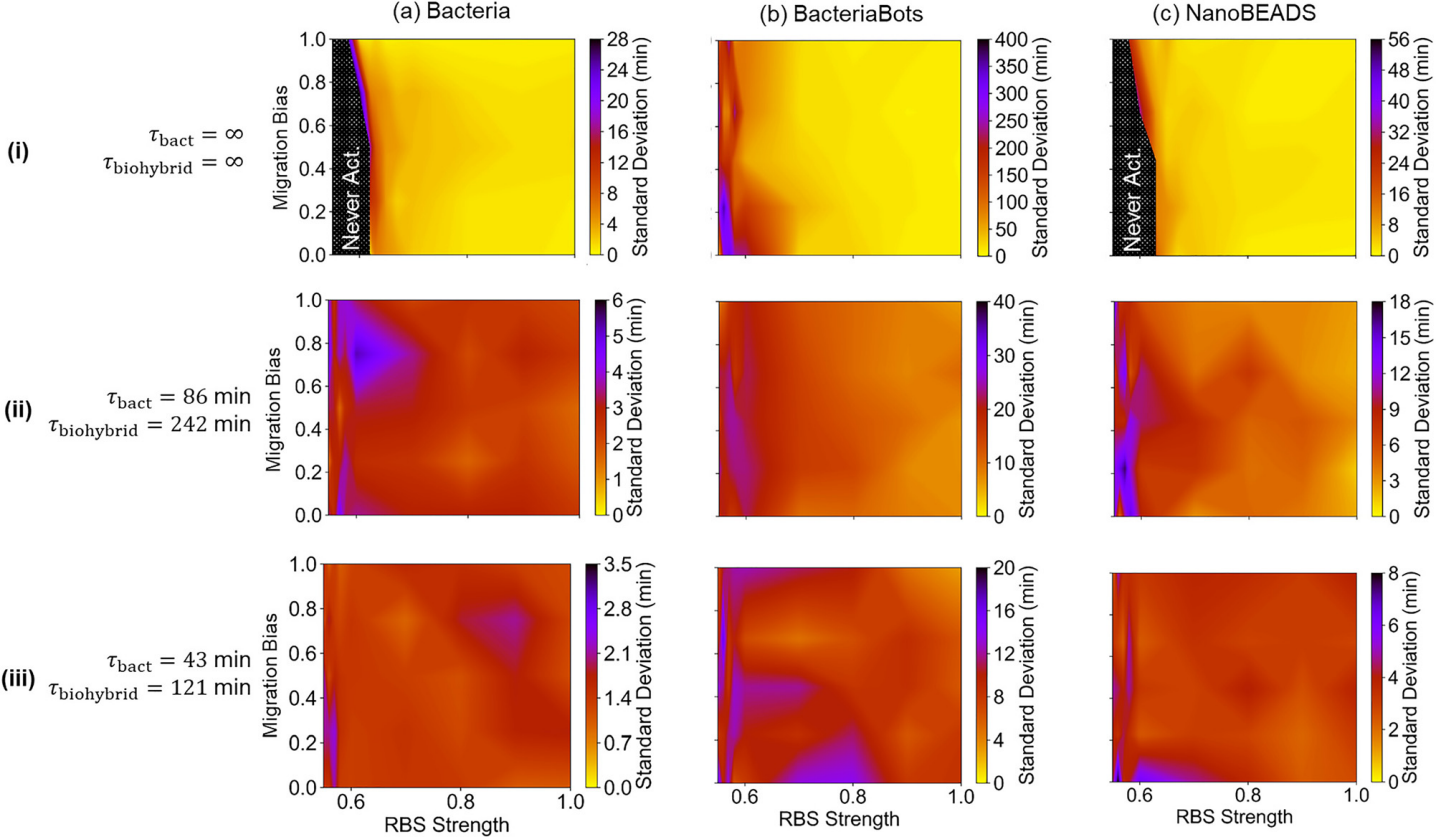
Robustness of emergent behavior across circuit sensitivity and migration bias design space. The standard deviation in simulated activation time as a function of the chemotaxis response (migration bias) and RBS strength (circuit sensitivity) for the free-swimming bacteria (a), BacteriaBots (b), and NanoBEADS (c) in the absence of growth (i), at half the experimentally measured growth rates (ii), and the experimentally measured growth rates (iii). Black shaded regions indicate zones where the agents reached a steady non-activated state.

Next, the robustness in QS response, assessed by quantitating the standard deviation in QS activation time, was investigated as a function of migration bias and RBS strength (Fig. 6). Smaller values and less variation across the parameter space indicate higher fidelity in achieving a designed activation time. In the absence of growth [Fig. 6(i)], each system is relatively robust for RBS designs of strength approximately 0.63 or higher. More relative variations occurred for simulations with growth. In all cases, more variation in activation time occurred at low RBS strengths and/or low migration biases. The plots for BacteriaBot simulations, shown in Fig. 6(b), are particularly insightful. While BacteriaBot activation time did not appear to significantly depend on migration bias [Fig. 5(b)], a clear role of chemotaxis in robustness is illustrated by its effect on standard deviation [Fig. 6(b)]. The fact that chemotaxis had a minimal role in robustness in the absence of growth [Fig. 6(b-i)] demonstrates that it is the chemotaxis of free bacteria derived from those attached to the microparticles, not the BacteriaBots themselves, that influence activation time robustness. Ascertaining unexpected results such as these highlights the important role simulation can play in robust system design.

### D. Decentralized control of microbial biohybrid systems for localized decision making

**FIG. 7.**
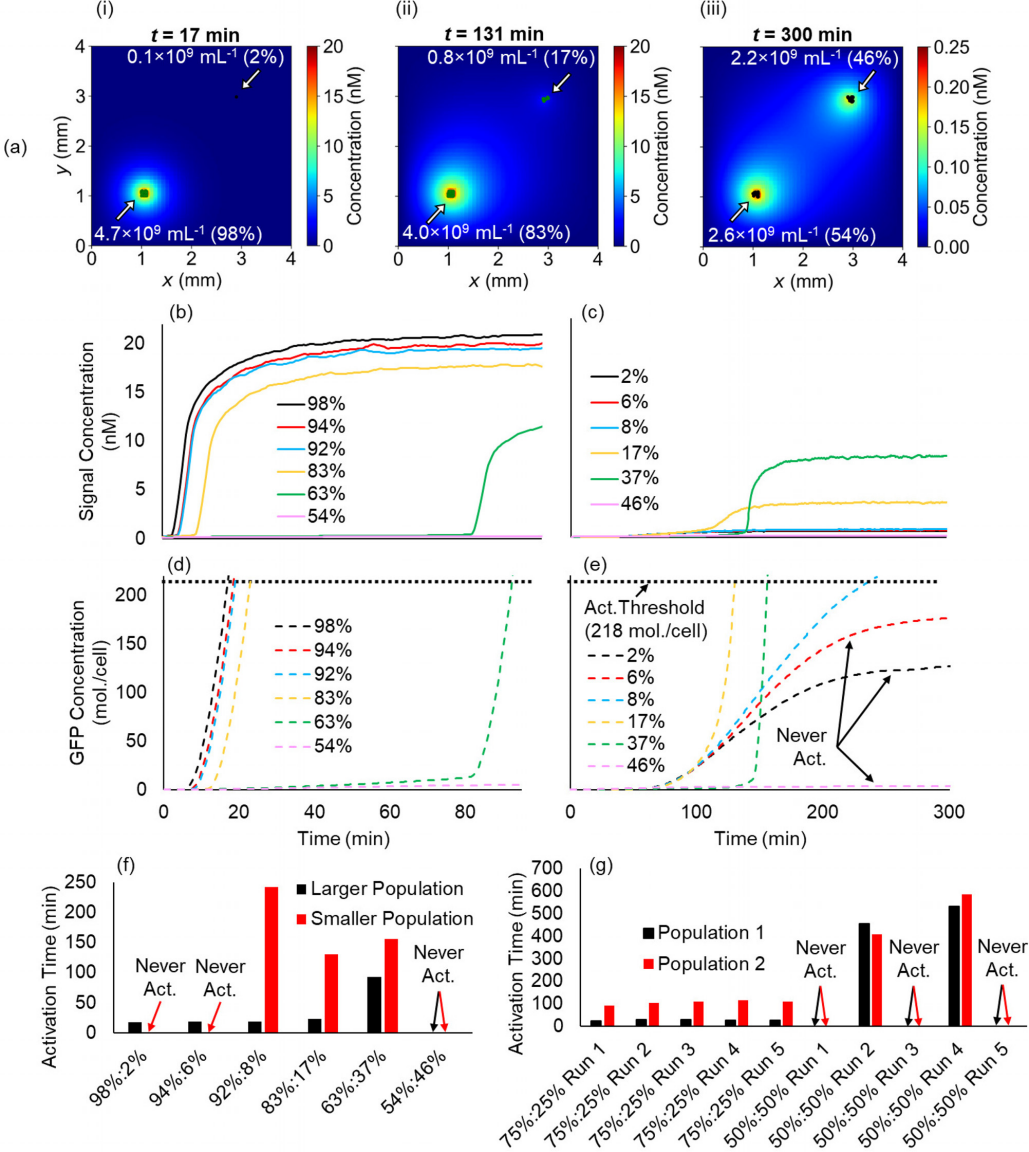
Localized decision making in distributed biohybrid swarms. (a) Snapshots of the signal concentration field for two populations of NanoBEADS seeded at various ratios leading to steady state activation (green) of the larger and steady state inactivation (black) of the smaller (i), steady state activation of both (ii), and steady state inactivation of both (iii), average signal concentration perceived by the agents of the larger (b) and smaller populations (c) vs time for each ratio (legend indicates percentage of total dose), average intracellular GFP concentration for agents of the larger (d) and smaller (e) populations vs time for each ratio, (f) the activation time for each population of agents for each ratio, and (g) activation time for each population of BacteriaBots seeded in an analogous scenario across five simulation replicates. The overall concentration of NanoBEADS and BacteriaBots contained an equivalent concentration of bacteria (4.8 × 10^9^ ml^−1^; 1 per NanoBEADS or 12 per BacteriaBot) in all scenarios.

Finally, we investigated the decentralized control of swarms of NanoBEADS and BacteriaBots as a mechanism to facilitate local decision making through QS activation at high population densities while precluding it in areas that do not become densely colonized. This would be crucial to our ability to design QS-based drug delivery systems that would become activated in the densely colonized tumor tissue but not in the surrounding normal tissue, for instance. To this end, we considered a hypothetical scenario wherein the same total number of NanoBEADS agents (an overall concentration of 0.3 × 10^7^ ml^−1^ in a 4 × 4 mm^2^ simulation domain, which amounts to 48 agents) densely colonize, without growth, two spatially separated 100 × 100 *μ*m^2^ regions within the domain at various ratios to represent a given dose being seeded in multiple locations [Fig. 7(a)]. Given that the QS signal considered in our system is the small molecule 3-oxohexanoyl-homoserine lactone (AHL, MW: 213 Da), we simulated signal transport with a diffusion coefficient reduced to 75% of its value in water (490 *μ*m^2^/s), as an estimated diffusion coefficient for AHL in the extracellular matrix. We found that two different stable states (i.e., activated and not-activated) would indeed occur given the appropriate population densities and sufficient separation between the two. For a separation distance of 2.7 mm, populations smaller than 8% of the total dose (<0.4 × 10^9^ ml^−1^) remained in a stable non-activated state. On the other hand, the signal concentration quickly rose to cause rapid local activation at the larger population consisting of a fraction of 92% or more of the population [>4.4 × 10^9^ ml^−1^, Figs. 7(b)–7(e)]. If the fraction of the total population present in the smaller population was increased to a critical amount of 8% or higher, it became activated following the activation of the larger population due to diffusion of the signal. As the fractions of the populations became more comparable, signal concentration increases and subsequent activation was further delayed in the larger population and reduced in the smaller population [Fig. 7(f)] until the low local concentration of each population precluded activation [Figs. 7(b)–7(e), magenta curves]. This is because the local concentration of each population was not high enough to produce signal at sufficient rates to overcome signal loss due to degradation and transport away from the agents [Fig. 7(a-iii)]. In contrast to NanoBEADS (with each agent comprising one bacterium), analogous simulations with BacteriaBots (each agent contains 12 bacteria on average) predict that two different but stable activation states cannot occur for the same 2.7 mm separation distance [Fig. 7(g)]. For four simulated BacteriaBot agents, a 3:1 separation ratio always resulted in activation of both populations. Populations at a 1:1 ratio (each concentrated to 2.0 × 10^8^ ml^−1^) however resulted in a highly stochastic outcome in which activation sometimes occurred and sometimes did not. In an analogous scenario, for more uniformly dispersed NanoBEADS (the two populations each at 2.4 × 10^9^ ml^−1^), activation would never occur for either.

**FIG. 8.**
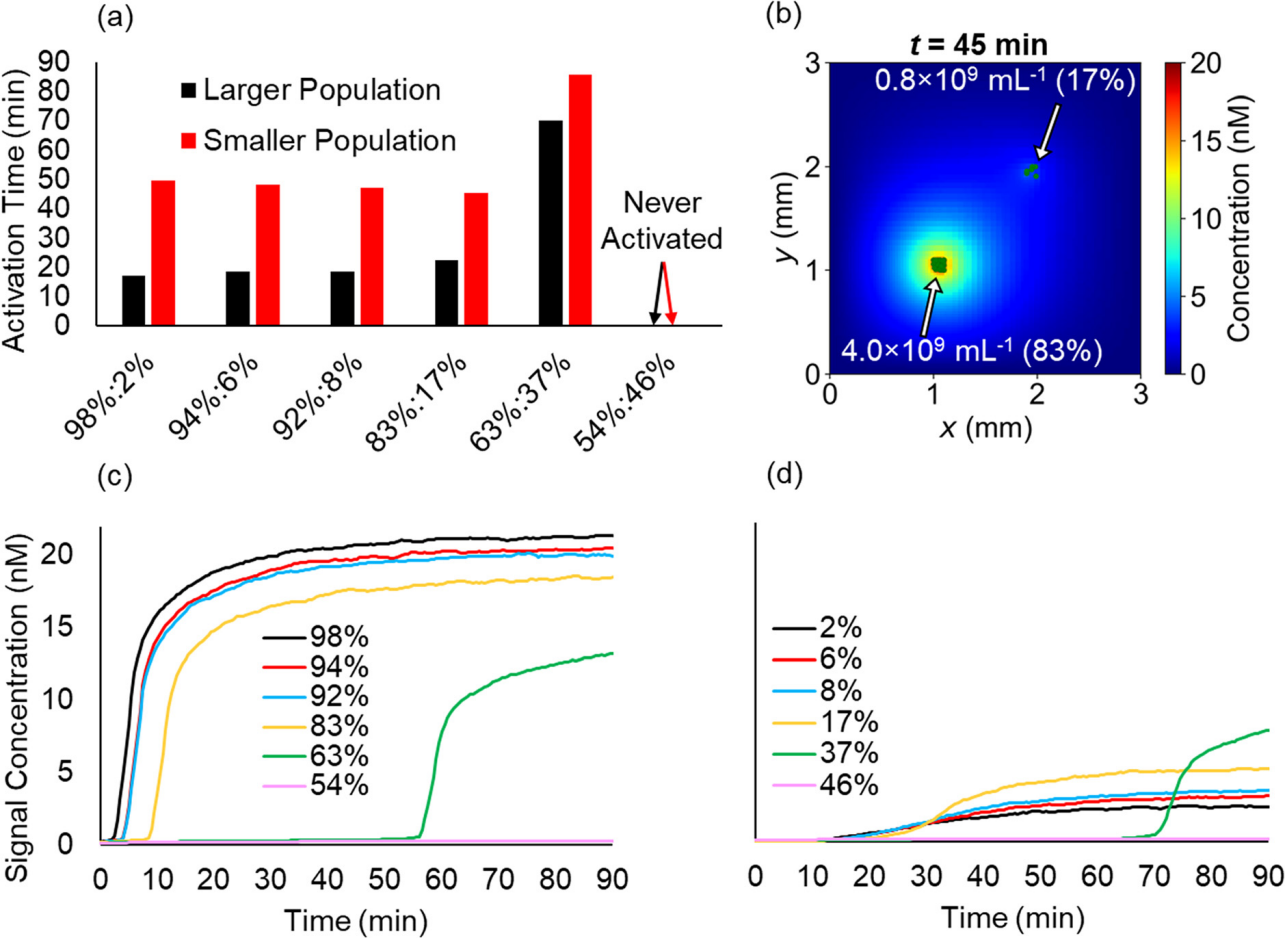
Decentralized control is robust across spatially separated populations in close proximity. (a) Activation time for each population of NanoBEADS separated by 1.7 mm, (b) a snapshot of the signal concentration field at the activation time for the 83%:17% ratio case, and average signal concentration perceived by the agents of the larger (c), and smaller (d) population fractions.

Overall, the robustness of activation of the entire population increased when the separation distance was decreased. When the same overall dose and concentration ratios discussed above were seeded at a reduced separation distance of 1.3 mm, activation occurred in both populations regardless of the ratio until the same critical low-density threshold as was found for the 2.7 mm separation distance was reached [54% and 46% of the total population in larger and small populations, respectively; Fig. 8(a)]. As long as one population was locally dense enough to become activated, signal transport dynamics allowed for activation of the smaller population [Figs. 8(b)–8(d)]. This further demonstrates the robustness of QS-based decentralized control. Although a target site may be colonized at several distinct locations by the administered dose, agents throughout the site will become activated due to their proximity. At the same time, other far away sites will remain inactive.

Altogether, our results demonstrate several key principles in biohybrid system design: (1) agents densely colonizing a target site (e.g., tumor) may perform their task without causing activation of sparse populations in off-target sites nearby, (2) the choice of agent dictates dose and robustness requirements, and (3) simulations are critical in illuminating non-intuitive results toward informed design with predictable outcomes.

## III. CONCLUSIONS

We have implemented a unique data-driven stochastic model for simulating the motility and chemotaxis of bacteria-based biohybrid agents based on limited amounts of experimental data. We integrated this computationally efficient model with an experimentally validated computational model of QS to simulate the emergent behavior in populations of biohybrid cargo-carrying agents, BacteriaBots and NanoBEADS, and compared the results to free-swimming engineered bacteria. We then investigated system robustness across genetic circuit sensitivity design space, offering a practical means for making informed biohybrid system design decisions. Finally, we showed how decentralized control can be an effective mechanism for causing desired activation at target sites, even when the agents are spread across several zones in close proximity, but that off-target sites colonized at low density would remain in a non-activated state. Overall, we showed that NanoBEADS offer comparable performance to free-swimming bacteria, making them a suitable choice as drug delivery agents when engineering bacteria for drug synthesis is not an option. BacteriaBot emergent behavior proved to allow for activation at lower equivalent bacterial concentrations than NanoBEADS, albeit more stochastic, although the agent size may be a limiting factor for some *in vivo* applications. In the future, our method of simulating agent motility could be applied to other motile biohybrid systems, and our model could be used for other biohybrid systems coupled with engineered QS bacteria.

## IV. METHODS

### A. Modeling motility and chemotaxis

The xy-coordinates in each time-lapse image of swimming NanoBEADS agents, recorded at approximately 14.1 frames per second (FPS) or 12.4 FPS, were tracked for approximately 6 s, and the coordinates of motile BacteriaBots were obtained from time-lapse images acquired at 1.3 FPS for 50 s. NanoBEADS were not tracked if the attached particles were not visible (assessed through fluorescence imaging) or if they physically interacted with another agent during the duration of the experiment. Likewise, BacteriaBots were not tracked if they interacted with another agent during the experiment. A total of 154 NanoBEADS tracks and 26 BacteriaBots tracks were analyzed. The velocity vector between every two successive data points was calculated and used to determine the rate of translation (speed) and the time rate of orientation change between each two velocity vectors [Fig. 2(c)]. These data were stored in sequence. For simulations, half of all experimental trajectories were randomly grouped together, while the remaining half was placed in a second group. For simulations that included a chemical gradient, agents that were oriented to translate toward an increasing concentration randomly chose a set of rates of orientation change and speeds derived from a single tracked agent from the first group and implemented these in its motile behavior. Likewise, agents facing a decreasing simulated chemical concentration were sampled from the second group. The particular speed and rate of orientation change for NanoBEADS agents were updated every 0.07 s (14 FPS) in accordance with experimental data, implementing the subsequent speed and rate of orientation change from the chosen trajectory. The speed and rate of orientation change for BacteriaBots were updated every 0.77 s (1.3 FPS) in accordance with experimental data. When any given simulated agent implemented every recorded speed and rate of orientation change of an experimental trajectory, another trajectory was randomly selected from the appropriate group, depending on the agent's orientation at that point in time.

In order to match experimental chemotaxis curves, data were randomly pooled 300 times, and simulations of 1000 agents were run for each particular separation of data. The binned data that gave the best match between experimental and simulated CPC at each point in time analyzed in experiments was chosen and used for subsequent simulations at that particular chemical gradient. For the robustness simulations used to produce Figs. 5 and 6, simulations of 1000 agents were run for 85 min, and the mean CPC for the final 20 min was taken as the steady state CPC. Particular sets of binned data were chosen to provide steady state CPCs ranging from 0 to 0.9, which corresponds the migration bias parameter ranging from 0 to 1.0 (i.e., a migration bias of 0 corresponds to a steady state CPC of 0, but a migration bias of 1.0 corresponds with a steady state CPC of 0.9).

The motility and chemotaxis of free-swimming bacteria, used for baseline comparison in this work, was based on our previous work.^57^ Briefly, the motility of each bacteria agent was modeled as periods of runs (i.e., linear continuous translations), interspersed with tumble periods (i.e., directional reorientations). The probability, p(τ), of a run or tumble event increases with time,

$$p\left(\tau \right)=\int_{0}^{\tau }\lambda_{i}e^{-\lambda_{i}t}dt,$$ (3)

where $\lambda_{i}^{-1}$ is the mean run or tumble duration, and τ is the particular duration.^51^ The detection of a gradient of a chemoeffector causes a biased random walk by altering the mean duration of the run period. Thus, the run duration depends on the spatial and temporal derivative of chemoeffector concentrations. The mean run time was modeled as^62^

$$\tau^{\pm }\left(\vec{r},t\right)=\tau_{0}\exp\left(\pm \sigma_{\mathrm{chemo}}\frac{DC}{Dt}\right),$$ (4)

where $\vec{r}=(x,y,z)$ is the location of the bacterium, $\tau_{0}$ is the average run duration in a chemically isotropic environment,^63^ $\sigma_{\mathrm{chemo}}$ is the chemotactic sensitivity, D/Dt is the material derivative, and C is the number of chemoreceptors to which chemoeffector molecules are bound. Michaelis-Menten-like receptor–ligand binding kinetics was assumed,

$$C\left(s\right)=\frac{C_{T}s}{K_{d}+s},$$ (5)

where $C_{T}$ is the total number of chemoreceptors for an agent, $s=s(\vec{r},t)$ is the local concentration of the chemoeffector, and $K_{d}$ is the dissociation constant for a chemoeffector binding to a receptor. From Eqs. (4) and (5), the mean run time is

$$\tau^{\pm }\left(\vec{r},t\right)=\tau_{0}\exp\left[\pm \sigma_{\mathrm{chemo}}\frac{{C_{T}K}_{d}}{{\left(K_{d}+s\right)}^{2}}\;\left(\frac{\partial s}{\partial t}+{\vec{V}}_{b}\cdot \nabla s\right)\right],$$ (6)

where ${\vec{V}}_{b}$ is the bacterium agent's velocity vector. For all chemoattractants (all simulations herein), $\tau =\tau^{+}$.

In simulations, each bacterium agent was assigned a run time following the end of a tumble period by using Eq. (6) to first calculate a mean run time, followed by Eq. (3) to modulate the run time for stochastic variability (with $\lambda_{i}^{-1}=\tau^{\pm }$). The orientation of a bacterium agent was changed during a tumble phase by randomly sampling from a log-normal distribution with a mean of $\theta_{\mu }$ and a variance of $\theta_{\sigma }^{2}.$^63^ The values used for parameters of the chemotaxis model are given in Table I.

**TABLE I.** Model parameters.

Parameter	Variable	Value	Source
Average run duration in the absence of a chemoeffector gradient	$\tau_{0}$	0.86 s	^63^
Average tumble duration	$\tau_{T}$	0.14 s	^63^
Chemotactic sensitivity for l-aspartic acid	$\sigma_{\mathrm{chemo}}C_{T}$	35 s	Estimated
Dissociation constant for l-aspartic acid	$K_{d}$	18 *μ*M	Estimated
Bacteria swimming speed	$\left\|\||{\vec{V}}_{b}\|\right\|$	34 *μ*m/s	Measured
Mean change in bacteria bearing between successive run phases	$\theta_{\mu }$	68°	^63^
Standard deviation of bacteria bearing change between run phases	$\theta_{\sigma }$	36°	^63^
Basal QS signal (AHL) generation rate	$A_{1}$	3.19 molecules/s	Fitted
Upregulated QS signal (AHL) generation rate	$A_{2}$	234 molecules /s	Fitted
Hill constant	H	2.5	^65^
QS signal (AHL) upregulation threshold	$Q_{0}$	1.87 nM	Fitted
Rate of GFP translation	$k_{tr}$	4.0 × 10^−1^ molecules/s	Fitted
Rate of GFP maturation	$k_{G_{m}}$	3.02 × 10^−3^ s^−1^	Estimated^69^
Maximum rate of GFP degradation	$k_{\deg}$	5.54 molecules/s	Estimated^70,71^
Half-maximum concentration for GFP degradation	$K_{m}$	6650 molecules/cell	Estimated^70^
Diffusion coefficient of the QS signaling molecule (AHL) in water	$D_{\mathrm{signal}}$	490 *μ*m^2^/s	^72^
Rate of QS signal (AHL) degradation	$R_{d}$	10.8% h^−1^	^73^

### B. Modeling physical interactions

For simplicity, the model assumes that all agents are spherical for the purposes of modeling collisions and preventing agent overlap. Bacteria and NanoBEADS were each modeled as 2 *μ*m-diameter spheres, while BacteriaBots were modeled as 6 *μ*m-diameter spheres. Collisions were modeled as inelastic and simply resulted in agent pause until a change in orientation caused the collision to end. Note that agent collisions were not highly prevalent in this work, as most of the simulated concentrations were very low.

### C. Modeling QS

We utilized our experimentally validated model of QS in this work.^57^ Briefly, the QS circuit is bi-stable with a “low” (non-activated) state and a “high” (activated) state. This can be modeled using a Hill function,^64,65^

$$A_{t}=\eta \left(A_{1}+A_{2}\frac{Q^{H}}{Q^{H}+Q_{0}^{H}}\right),$$ (7)

where $A_{t}$ is the total rate of signaling molecule production, $A_{1}$ and $A_{2}$ are the constitutive and upregulated rates of signal generation, respectively, $Q=Q(\vec{r},t)$ is the local concentration of the signal, $Q_{0}$ is the upregulation threshold concentration, and H is the Hill constant, which indicates how quickly the system transitions from its low state to its high state after being exposed to a critical concentration, Q. The circuit sensitivity is η, which we defined as the predicted translation initiation rate (TIR) of an RBS sequence relative to the predicted TIR of a reference RBS.^66,67^ In our experimental system, we placed the *gfpmut3b* gene for green fluorescent protein (GFP) with an *lva* degradation tag (BBa_J04031) downstream of the *lux* QS promoter to serve as a proxy for QS activation. In practice, another gene of practical interest for the target application could be used to replace *gfpmut3b*. We adopted the coupled system of differential equations presented in Ref. 68 to model the intracellular kinetics of immature GFP ($G_{i}$) translation and its maturation into its fluorescent form $G_{m}$:

$$\frac{dG_{i}}{dt}=k_{\mathrm{tr}}\frac{Q^{H}}{Q^{H}+Q_{0}^{H}}-k_{G_{m}}G_{i}-\mu G_{i}-k_{\deg}\frac{G_{i}}{G_{i}+G_{m}+K_{m}}$$ (8)

and

$$\frac{dG_{m}}{dt}=k_{G_{m}}G_{i}-\mu G_{m}-k_{\deg}\frac{G_{m}}{G_{i}+G_{m}+K_{m}},$$ (9)

where $k_{\mathrm{tr}}$ is the maximum rate of production of immature GFP, $k_{G_{m}}$ is the rate of maturation of $G_{i}$ into $G_{m},$^69^ μ is the bacterial agents' (e.g., bacteria, NanoBEADS, or bacteria attached to a microparticle) growth rate, $k_{\deg}$ is the maximum rate of protease-mediated degradation,^70,71^ and $K_{m}$ is the concentration of GFP at which the rate of degradation is half its maximum rate (kinetics was assumed to be the same for both $G_{i}$ and $G_{m}$).

Signal transport and degradation in the extracellular environment are governed by a diffusion–reaction equation,

$$\frac{\partial Q}{\partial t}=\nabla \cdot \left(D_{\mathrm{signal}}\nabla Q\right)-R_{d}Q,$$ (10)

where $D_{\mathrm{signal}}$ is the diffusion coefficient of the signal^72^ and $R_{d}$ is the rate of signal degradation.^73^ Note that we have shown that advection is insignificant relative to the rate of transport via diffusion of the small AHL signaling molecules (Péclet Number $\mathrm{Pe}=\frac{L_{c}\left\|{\vec{V}}_{b}\right\|}{D_{\mathrm{signal}}}\approx 0.28$); thus, advective transport was not modeled.^57^ All parameters for the QS model are given in Table I.

### D. Modeling growth

Growth was modeled by assigning timers to each agent at the start of a simulation, each measuring the time until that agent should double. For NanoBEADS and free-swimming bacteria, a daughter agent of the same type (i.e., a NanoBEADS agent or bacterium agent) was created at the location of the mother agent. For BacteriaBots, we assumed that the attached bacteria doubled at the same rate as NanoBEADS agents, but the daughter agent became a free-swimming bacterium agent, thus also adopting the growth rate of free-swimming bacteria. Doubling times were implemented as the experimentally measured values of 43 min and 121 min for free-swimming bacteria and NanoBEADS, respectively, unless otherwise indicated.^74^ The initial doubling time of agents at the start of the simulation was made stochastic by randomly sampling from a uniform distribution ranging from 0 to $\tau_{\mathrm{dbl}}$, where $\tau_{\mathrm{dbl}}$ is the input doubling time for each agent type. Once an agent produced a daughter cell, its growth timer was reset to $\tau_{\mathrm{dbl}}$.

### E. Experiments

#### 1. BacteriaBot assembly and microfluidic chemotaxis experiments

Experimental methods followed were similar to the methods described in a prior work.^32^ Briefly, motile isolates of *E. coli* MG1655 harboring a plasmid (pHC60) for green fluorescent protein (GFP) expression were cultured at 32 °C and 150 RPM overnight in tryptone broth (1% w/v tryptone, 0.5% w/v sodium chloride) supplemented with 10 *μ*g/ml tetracycline. The bacteria were diluted 100-fold and grown until an OD_600_ of 0.5 was reached. The bacteria were then harvested and resuspended in a formulation of motility medium that provided neutral BacteriaBot buoyancy (0.01 M potassium phosphate, 0.067 M sodium chloride, 10^−4^ M EDTA, 0.21 M glucose, and 0.002% Tween-20, pH = 7.0) and supplemented with biotin-labeled goat polyclonal anti-lipid A lipopolysaccharide (LPS) antibody (Thermo Scientific, Waltham, MA) at 1 *μ*g/ml. Spherical carboxylate polystyrene particles (Polysciences, Warrington, PA) of 6 *μ*m-diameter were washed with 30% isopropanol and suspended in motility buffer supplemented with 5 *μ*g/ml streptavidin-Cy3 (Sigma-Aldrich, St. Louis, MO). Each solution was incubated separately for at room temperature for 1 h while vortex mixing at 500 RPM. The two were then combined and vortex-mixed for another 30 min. All BacteriaBot experiments were performed in the neutrally buoyant motility medium to limit changes in the vertical location of the agents.

Experiments were performed using a microfluidic device with three parallel channels in polyethylene glycol diacrylate (PEG-DA) gel.^75^ For chemotaxis experiments, a quasi-linear gradient of 1.7 × 10^−5^ M/mm l-aspartic acid (chemoattractant) was established in motility medium by flowing motility buffer through one outer channel and a solution of 33.8 *μ*M concentration through the other outer channel, causing the l-aspartic acid to diffuse across the center channel containing BacteriaBots. To gather the data tracked and used for the motility model, isotropic experiments were performed with motility buffer flowed through each side channel.

#### 2. NanoBEAD assembly and microfluidic chemotaxis experiments

NanoBEADS were assembled based on reported methods.^56^ *E. coli* MG1655 was used in all experiments.^76^ Bacteria cultures were incubated overnight in 10 ml of fresh Luria-Bertani (LB) Broth (1% w/v of tryptone, 0.5% w/v of NaCl, and 0.5% w/v of yeast extract) at 30 °C and 150 RPM. A 100 *μ*l volume of the dense overnight culture was used to inoculate 10 ml fresh LB, and the bacteria were grown to an OD_600_ of 0.5. The bacteria were harvested (1 ml) and centrifuged at 1700×*g* at room temperature for 5 min and resuspended in 1 ml of motility buffer [0.01 M potassium phosphate, 0.067 M sodium chloride, 10^−4^ M EDTA, 0.01 M glucose, and 0.002% (v/v) Tween-20]. The bacteria were twice washed in this motility buffer before being incubated with biotin-conjugated goat polyclonal anti-lipid A LPS antibody (Thermo Scientific, Waltham, MA) at 10 *μ*g/ml. This suspension of bacteria and antibody was mixed using a vortex shaker for 1 h at 600 RPM to promote robust antibody labeling of each bacterial cell. The labeled bacterial suspension was then centrifuged at 1700×*g* for 5 min to remove the excess antibody from the solution, and the bacteria were concentrated into 50 *μ*l of motility buffer. Streptavidin-coated carboxylate polystyrene nanoparticles (109 nm diameter and 390 nm diameter, Bangs laboratories, Fishers, IN) were agitated with biotinylated antibody-coated bacteria at various ratios for 30 min. Thus, NanoBEADS were constructed through the formation of streptavidin-biotin bonds between the streptavidin-coated nanoparticles and biotin-conjugated antibody-labeled bacteria. The antibody used in this work was raised against the “O” antigens present only on the outer membrane of the bacteria, restricting the attachment of the antibody (and thus streptavidin coated nanoparticles) to the cell surface. To acquire data tracked and used for the motility model, assembled NanoBEADS were suspended in a thin film between two #1 coverslips. Microscopy images and time-lapse videos of the NanoBEADS were captured using a Zeiss AxioObserver Z1 inverted microscope equipped with an AxioCam mRM camera and a 63× oil objective.

For microfluidic chemotaxis experiments, devices were fabricated according to the methods in Sec. IV E 1. Only 390 nm nanoparticles were used for these experiments, and particles and bacteria were combined at a 100:1 ratio. A 5.0 × 10^−4^ g mL^−1^ mm^−1^ gradient of casamino acids was established spanning a 500 *μ*m-wide microfluidic channel to induce chemotactic migration.

### F. Ethics approval

No ethics approval was required for this work.

## SUPPLEMENTARY MATERIAL

See the supplementary material for the results of simulations using rates of orientation change and speeds sampled randomly rather than in the sequence in which they occurred. Figure S1 shows plots of persistence vs time [similar to those shown in Figs. 2(d)–2(i)] for these randomly sampled data.
